# Genomic Study of *Babesia bovis* Infection Level and Its Association With Tick Count in Hereford and Braford Cattle

**DOI:** 10.3389/fimmu.2020.01905

**Published:** 2020-08-28

**Authors:** Ligia Cavani, Camila Urbano Braz, Rodrigo Giglioti, Cintia Hiromi Okino, Claudia Cristina Gulias-Gomes, Alexandre Rodrigues Caetano, Márcia Cristina de Sena Oliveira, Fernando Flores Cardoso, Henrique Nunes de Oliveira

**Affiliations:** ^1^School of Agricultural and Veterinary Sciences, São Paulo State University (Unesp), Jaboticabal, Brazil; ^2^Empresa Brasileira de Pesquisa Agropecuária, Embrapa Pecuária Sudeste, São Carlos, Brazil; ^3^Empresa Brasileira de Pesquisa Agropecuária, Embrapa Pecuária Sul, Bagé, Brazil; ^4^Empresa Brasileira de Pesquisa Agropecuária, Embrapa Recursos Genéticos e Biotecnologia, Brasília, Brazil

**Keywords:** babesiosis, cross-validation, genetic parameters, genomic selection, genome-wide association studies

## Abstract

Bovine babesiosis is a tick-borne disease caused by intraerythrocytic protozoa and leads to substantial economic losses for the livestock industry throughout the world. *Babesia bovis* is considered the most pathogenic species, which causes bovine babesiosis in Brazil. Genomic data could be used to evaluate the viability of improving resistance against *B. bovis* infection level (IB) through genomic selection, and, for that, knowledge of genetic parameters is needed. Furthermore, genome-wide association studies (GWAS) could be conducted to provide a better understanding of the genetic basis of the host response to *B. bovis* infection. No previous work in quantitative genetics of *B. bovis* infection was found. Thus, the objective of this study was to estimate the genetic correlation between IB and tick count (TC), evaluate predictive ability and applicability of genomic selection, and perform GWAS in Hereford and Braford cattle. The single-step genomic best linear unbiased prediction method was used, which allows the estimation of both breeding values and marker effects. Standard phenotyping was conducted for both traits. IB quantifications from the blood of 1,858 animals were carried using quantitative PCR assays. For TC, one to three subsequent tick counts were performed by manually counting adult female ticks on one side of each animal's body that was naturally exposed to ticks. Animals were genotyped using the Illumina BovineSNP50 panel. The posterior mean of IB heritability, estimated by the Bayesian animal model in a bivariate analysis, was low (0.10), and the estimations of genetic correlation between IB and TC were also low (0.15). The cross-validation genomic prediction accuracy for IB ranged from 0.18 to 0.35 and from 0.29 to 0.32 using k-means and random clustering, respectively, suggesting that genomic predictions could be used as a tool to improve genetics for IB, especially if a larger training population is developed. The top 10 single nucleotide polymorphisms from the GWAS explained 5.04% of total genetic variance for IB, which were located on chromosomes 1, 2, 5, 6, 12, 17, 18, 16, 24, and 26. Some candidate genes participate in immunity system pathways indicating that those genes are involved in resistance to *B. bovis* in cattle. Although the genetic correlation between IB and TC was weak, some candidate genes for IB were also reported in tick infestation studies, and they were also involved in biological resistance processes. This study contributes to improving genetic knowledge regarding infection by *B. bovis* in cattle.

## Introduction

Bovine babesiosis is a tick-borne disease caused by intraerythrocytic protozoa of the *Babesia* genus leading to substantial economic losses for the livestock industry throughout the world (1–3). In Brazil, bovine babesiosis is caused by *Babesia bovis* and *Babesia bigemina*, which are exclusively transmitted by the one-host tick *Rhipicephalus microplus* (4, 5). *B. bovis* is the most pathogenic species (6). The infective forms, which are in tick saliva, invade the host's erythrocytes, multiply until hemolysis, and invade new erythrocytes until the host dies or develops immunity (7). Calves have an innate age-related resistance to babesiosis. Thus, in regions where there is endemic stability, calves are exposed to babesiosis and develop immunity to the disease (6). On the other hand, in regions of endemic instability, where climatic conditions prevent the survival of ticks during a certain period of the year, calves may not be infected when they are young, and outbreaks of babesiosis may occur when ticks reinfest the pastures (8).

Bock et al. (9) and Jonsson et al. (10) observed that zebu (*Bos taurus indicus*) were more resistant to *B. bovis* than taurine (*Bos taurus taurus*) breeds. More recently, the levels of *B. bovis* infection in bovine blood samples have been successfully quantified through quantitative PCR (qPCR) assays (11–14). Differences in these levels were observed between zebu (*B. taurus indicus*) and taurine (*B. taurus taurus*) breeds (12), corroborating previous findings. Phenotypic variation of the level of *B. bovis* infection has been reported (12, 15). However, no quantitative genetic studies have been found in the literature, and little is known regarding its association with tick resistance.

Advances in molecular genetic techniques have allowed the incorporation of genetic markers such as single nucleotide polymorphisms (SNPs) into the breeding analysis, enabling earlier accurate predictions (16). Genomic selection is a powerful strategy to increase the rate of genetic gain, especially in traits where selection based on phenotypic records is difficult, such as disease resistance traits and low heritability traits (17, 18). Cardoso et al. (19) showed that it is possible to control tick infestation through the genomic selection of tick-resistant animals. Therefore, the genetic improvement could be an important tool for *B. bovis* infection control. Moreover, using SNP information allows the detection of genomic regions associated with *B. bovis* infection level through genome-wide association studies (GWAS) (20) and, thus, contributes to a better understanding of the genetic basis of this economically important and complex trait. Regarding a tick resistance trait in cattle, many studies have identified genomic regions through association studies (21–26).

The objective of this study was to estimate the genetic correlation between *B. bovis* infection level (IB) and tick count (TC), evaluate predictive ability and application of genomic selection, and perform GWAS for IB in Hereford and Braford cattle to better understand the biological mechanisms underlying IB and its association with tick resistance.

## Materials and Methods

### Phenotype Data

The data set was provided by the Delta G Connection breeding program (Gensys Associated Consultants, Porto Alegre, RS, Brazil), which included Hereford and Braford cattle raised on pastures in southern Brazil. The Braford breed is a combination of 3/8 indicine breeds and 5/8 Hereford; however, in Brazil, the breeders are allowed to vary the relative proportion of the component breeds. In addition to phenotypic records on IB and TC, pedigree information for the last three generations and genotype data were included. A total of 5,867 (1,915 Hereford and 3,952 Braford) animals provided TC records, and 1,858 animals (225 Hereford and 1,633 Braford) provided IB records, between the years 2010–2013. The average age of the animals during the evaluation period was 17.5 months (10.9–23.1 months).

#### *Babesia bovis* Infection Level

The IB was assessed by determining the number of copies of *B. bovis* target DNA sequence (cytochrome b gene). For that, DNA was extracted from blood samples of each animal collected on FTA cards using the Gensolve DNA recovery kit (Gentegra, Pleasanton, CA, USA). The concentration and quality of this DNA were determined in a NanoDrop spectrophotometer (NanoDrop Technologies Inc., Wilmington, Delaware, USA). The DNA samples were kept at −80°C until further analysis. After that, the qPCR was performed using the CFX^TM^ Real-Time PCR Detection System (BioRad, Hercules, CA, USA), according to Giglioti et al. (14). The primers cbosg 1 (F): 5′ -TGTTCCTGGAAGCGTTGATTC-3′ and cbosg 2 (R): 5′-AGCGTGAAAATAACGCATTGC-3′ amplify an 88-bp fragment from the cytochrome b gene of *B. bovis* (11, 27). The standard curves were plotted using 10-fold dilutions of synthetic DNA gBlocks® Gene Fragments (IDT, Coralville, IA, USA), which contain known concentrations of the *B. bovis* target sequence. To estimate the number of copies of the target DNA sequence (NC), the samples and controls were submitted to qPCR tests together with dilutions of synthetic DNA gBlocks®. Then, using software native to the CFX96 system, NC values for dilutions were utilized as a reference for the estimation of NC in the samples. Default settings were used for all parameters, except for the threshold line that was set to the same value, at 200 relative fluorescence unit, for all qPCR tests. All samples and controls were tested in duplicate, and samples with a standard deviation >0.5 were retested. Samples presenting NC > 0 and specific temperature melting (Tm) (77.5 ± 0.5°C) were considered positive.

#### Tick Count

The animals were naturally exposed to ticks, and after weaning, when the visual estimate of the average infestation across all animals in a management group (animals raised together, receiving the same feeding and sanitary management) exceeded about 20 engorged female ticks, counts were performed by manual counting adult female ticks (4.5–8 mm in length) on the right side of each animal (28). This process was carried out one to three times in each management group. Tick counts were performed in late spring, summer, and early fall, and the minimum period between counts was 30 days.

For the analyses, the IB and TC records were transformed in log10(x + 1) due to normality assumptions of the models used in this study. The descriptive statistics are shown in Table 1.

**Table 1:** Descriptive statistics for *Babesia bovis* infection level (IB) and tick counts (TC) in Braford and Hereford cattle.

**Traits**	**N^a^**	**Mean^**b**^**	**SD^**b**^**	**Minimum**	**Median^**b**^**	**Maximum^**b**^**
IB	1,858	719.9 (1.6)	5,920.68 (1.14)	0	79.2 (1.9)	154,199.5 (5.2)
TC	13,874	38.9 (1.4)	48.46 (0.47)	0	25 (1.4)	600 (2.8)

a *Number of records*.

b *Log-transformed scale data is shown in parentheses*.

#### Genotype Data

A total of 4,496 animals were genotyped with the Illumina BovineSNP50 BeadChip (50K; Illumina, San Diego, CA). Genotype quality control was performed using the R *snpStats* package (29) to remove samples with a call rate < 0.90, heterozygosity 3.0 SD above or below the observed mean, mismatching sex, and duplicated records. Only SNPs mapped to the autosomes, with call rates > 0.98, minor allele frequencies > 0.03, and not in a highly significant deviation from the Hardy–Weinberg equilibrium (*P* > 10^−7^), were considered for the analyses. Additionally, only the SNP with the highest minor allele frequency was retained when two SNPs were highly correlated (*r* > 0.98). After quality control, 39,919 SNP markers and 4,388 samples remained for the statistical analysis.

### Statistical Models

The genetic parameter estimations for IB and TC were performed by Bayesian inference in a bivariate analysis using an animal model. The Bayesian approach was chosen because the sample size was not large, and, to our knowledge, this is the first quantitative genetic study for IB, which is a new phenotype. The advantage of Bayesian methods, in this case, is that interpretation of the results and uncertainties about the estimates are facilitated, as all results are presented in terms of probabilities (30).

The model can be represented as follows:

$$\text{y}=\text{X}\beta +{\text{Z}}_{1}\text{a}+{\text{Z}}_{2}\text{p}+\text{e}$$

with the joint distribution of vectors **a**, **p**, and **e** as:

$$\begin{array}{l} \left[\begin{array}{c} \text{a}\\ \text{p}\\ \text{e} \end{array}\right]\sim \text{N}\left\{\left[\begin{array}{c} 0\\ 0\\ 0 \end{array}\right],\left[\begin{array}{ccc} {\text{G}}_{0}⊗\text{H} & 0 & 0\\ 0 & {\text{P}}_{0}⊗\text{I} & 0\\ 0 & 0 & {\text{R}}_{0}⊗\text{I} \end{array}\right]\right\}, \end{array}$$

where **y** is a vector of observations; **β** is a vector of systematic (fixed) effects; **a** is a vector of random additive genetic direct effects; **p** is the vector of random permanent environmental effects; **e** is a vector of random residuals; **X**, **Z**_**1**_, and **Z**_**2**_ are known incidence matrices; **G**_**0**_ and **P**_**0**_ are the additive genetic direct and environmental permanent effects (co)variance matrices, respectively; **H** is the additive genetic relationship matrix; **R**_**0**_ is the residual (co)variance matrix; and **I** is an identity matrix with suitable dimensions. Only the permanent environmental effects were included in the model for TC; therefore, **p**, **Z**_**2**_, and **P**_**0**_ were not considered for IB.

The **H** matrix combines genotype and pedigree information (31, 32), and its inverse (**H**^**−1**^) can be described in matrix notation as:

$${\text{H}}^{-1}={\text{A}}^{-1}+\left[\begin{array}{cc} 0 & 0\\ 0 & {\text{G}}^{-1}-{\text{A}}_{22}^{-1}\\ \; & \; \end{array}\right],$$

where **G** is a genomic relationship matrix constructed as in VanRaden (33) using current allele frequencies, and **A**_**22**_ is a numerator relationship matrix only for genotyped animals.

Concerning the systematic effects, contemporary groups (CGs) were included for IB and TC, as well as the effect of racial composition (zebu proportion, heterozygosity, and recombination loss computed from pedigree information). Linear and quadratic effects of animal age were considered only for TC. For IB, the linear effect of total DNA concentration available for qPCR assays was considered. The CG was composed of the animal from the same farm, sex, year and season of birth, and management group. For TC, the date of the phenotypic evaluations was also included in the CG. CGs with less than three observations were excluded from the data set. The total numbers of CGs for IB and TC were 15 and 227, respectively.

The GIBBS2F90 program (34) was used to obtain samples from the posterior distributions of genetic, permanent environmental, and residual (co)variances. A Gibbs sampling chain with 500,000 samples was generated, with the initial 50,000 samples discarded as burn-in based on visual inspection of trace plots and the convergence tests of Gelman and Rubin (35) and Geweke (36) as well as of Heidelberg and Welch (37) using the *coda* package (38) of the R software (39). The posterior distributions of the variance and covariance components were approximated based on the remaining 450,000 samples.

Genomic selection and GWAS were performed just for IB using the single-step genomic best linear unbiased prediction (ssGBLUP) approach, and the effects considered in the model were the same as previously described for the IB trait. The ssGBLUP method allows estimation of both breeding values and marker effects and combines genomic and pedigree relationships using the **H** matrix, as described earlier.

To estimate the genomic selection accuracy, cross-validation was applied. Furthermore, two animal grouping methods were used: k-means and random. More details are described later.

The predictive ability of genomic selection for IB was assessed by cross-validation, where 1,855 animals (1,631 Braford and 224 Hereford) with genotypes and IB phenotypes were divided into three groups by two strategies using R software (39). The strategies to divide the groups were k-means clustering of marker relationship distances and replicated 10 times at random. Average genomic relationships of each animal with all others within and between groups were calculated to characterize relatedness between training and validation sets (40). For each grouping strategy, 3-fold cross-validations were performed by alternately using records of two groups as training sets to derive genomic predictions for the third (validation) group, whose data were omitted in the analyses for marker effect estimation. Prediction accuracies, within a cluster *c*, were estimated as the correlation between predicted ($\hat{a}$) and estimated true (**a**) breeding values (${\hat{r}}_{\text{a}\hat{\text{a}}c}$), as proposed by Legarra et al. (41):

$${\hat{r}}_{\text{a}\hat{\text{a}}c}=PA/\sqrt{h^{2}}$$

in which PA is the predictive ability defined as the correlation between IB of animals from group *c* adjusted for the fixed effects and predicted values from cross-validation, represented by direct genomic value. Moreover, *h*^2^ is the heritability for IB.

The GWAS for IB were performed using the method proposed by Wang et al. (42), which is based on the ssGBLUP. The effects of the SNPs ($\hat{u}$) were obtained using the equation described as:

$$\hat{\text{u}}=\lambda \text{D}Z^{\prime }{\text{G}}^{*-1}{\hat{\text{a}}}_{\text{g}}$$

where $\hat{u}$ is the vector of estimated SNP effects; λ is the variance ratio calculated according to VanRaden et al. (43); ${\hat{a}}_{g}$ is the animal effect of genotyped animals; **Z′** is a transpose matrix that relates the genotypes of each locus; G^*^ is the weighted genomic relationship matrix; **D** is a diagonal matrix of the weights of SNP variances obtained by the algorithm with the following steps, where *t* is an iteration number, *p* is the allele frequency of the second allele, and *i* is the *i-th* SNP:

1. t = 0; **D**_(t)_ = **I**; **G**_(t)_ = **ZD**_(*t*)_**Z′**λ
2. Compute ${\hat{a}}_{g}$ by ssGBLUP;
3. Calculate $\hat{u}$_(t)_ = λ**D**_(t)_**Z′**${\text{G}}_{\left(\text{t}\right)}^{-1}$${\hat{a}}_{g}$;
4. Calculate the weight for each SNP: $d_{i_{\left(t+1\right)}}^{*}$= ${\hat{u}}_{i_{\left(t\right)}}^{2}$*2p*_*i*_*(1 – p*_*i*_*)* (44);
5. Normalize **D**_(t+1)_ = (tr(**D**_(0)_)/tr(D^*^_(t+1)_))D^*^_(*t*+1)_;
6. Calculate **G**_(t+1)_ = **ZD**_(*t*+1)_**Z′**λ;
7. Loop to step 3 for 3 times.

The analyses were carried out using the BLUPF90 family of programs (34). The results of GWAS are reported as the proportion of variance explained by a single SNP. A Manhattan plot was created using the R package “ggplot2” (45). Once the SNPs that explain the largest amount of IB genetic variance were identified, they were assigned to the candidate genes. The candidate genes were identified through the Ensembl genome database project, available at https://useast.ensembl.org/index.html, based on the *Bos taurus* ARS.120 reference assembly. For that, the genomic coordinates were expanded by 500 kb upstream and downstream; in this sense, an SNP was assigned to a candidate gene if it was located within or near to the gene. Gene ontology and biological pathway annotations of the genes were retrieved using the biomaRt package (46) and Reactome Pathway Knowledgebase (47), respectively.

## Results and Discussion

### Genetic Parameters

Estimates of heritability and repeatability for TC were low (0.127) and moderate (0.267), respectively (Table 2). The proportion of phenotypic variance explained by genetic variance of TC evaluated in the population of Hereford and Braford cattle was lower than other studies in Brazil with the same breeds that reported a heritability of 0.19 (19, 48). These differences between estimated heritability can be explained by population sample differences and by the model. The main model difference is the matrix relationships; in this study, we used genomic information (SNPs) to build the matrix relationships (H matrix). Lower estimates of genetic variance based on genomic relationships compared with those using pedigree relationships may occur; however, the estimates based on genomic relationships are frequently more accurate (49). Further, higher heritabilities would be expected in experimental stations' environmental conditions, as shown by Burrow (50), who reported a heritability of 0.42 for TC in a composite breed, as the conditions of the collection are more controlled. In the present study, the tick counts were carried out through collection by several technicians, and although all were trained for collection, this can be a factor in increasing experimental error. From this perspective, Ayres et al. (51) estimated heritabilities of 0.12 and 0.11 for TC in Brazilian Nellore × Hereford crossbred cattle, and Budeli et al. (52), in South African Bonsmara breed cattle, found heritabilities ranging from 0.03 to 0.17 in groups of animals divided according to mean tick count.

**Table 2:** Posterior mean and 95% highest posterior density intervals (within parentheses) of (co)variance components for *Babesia bovis* infection level (IB) tick counts (TC), and genetic correlation between IB and TC in Braford and Hereford cattle performed by Bayesian animal model in bivariate analysis.

**Parameter**	**IB**	**TC**
Additive genetic variance, σ^2^_a_	0.088 (0.040, 0.141)	0.012 (0.009, 0.016)
Permanent environmental variance, σ^2^_p_	–	0.014 (0.010, 0.071)
Residual variance, σ^2^_e_	1.048 (0.168, 0.890)	0.072 (0.070, 0.074)
Heritability, h^2^	0.077 (0.037, 0.124)	0.127 (0.093, 0.160)
Repeatability, r^2^	–	0.267 (0.245, 0.289)
Genetic correlation, r_IB,TC_	0.152 (−0.147, 0.445)	

Despite the low posterior mean for IB heritability (0.077), there is additive genetic variability for this trait, and therefore, selection responses may be obtained. No previous information on quantitative genetics for IB was found in the literature; however, low to moderate values for repeatability of IB in Angus (13) and Canchim (14) have been reported. Usually, the heritability estimates for disease traits are low mainly because of the complexity behind these phenotypes (53, 54). Moreover, the genetic correlation between IB and TC was weak (0.152). This suggests that selection for TC would not change IB considerably in the population of this study. Although no quantitative genetic studies were found for levels of babesiosis infection in cattle, Giglioti et al. (14) found the phenotypic correlation between tick count and *B. bovis* levels ranging from 0.02 to 0.17, in different ages. It is important to note that the number of records for IB is much lower than for TC; besides, IB is a new phenotype related to a disease.

### Genomic Selection for *Babesia bovis* Infection Level

**Figure 1:**
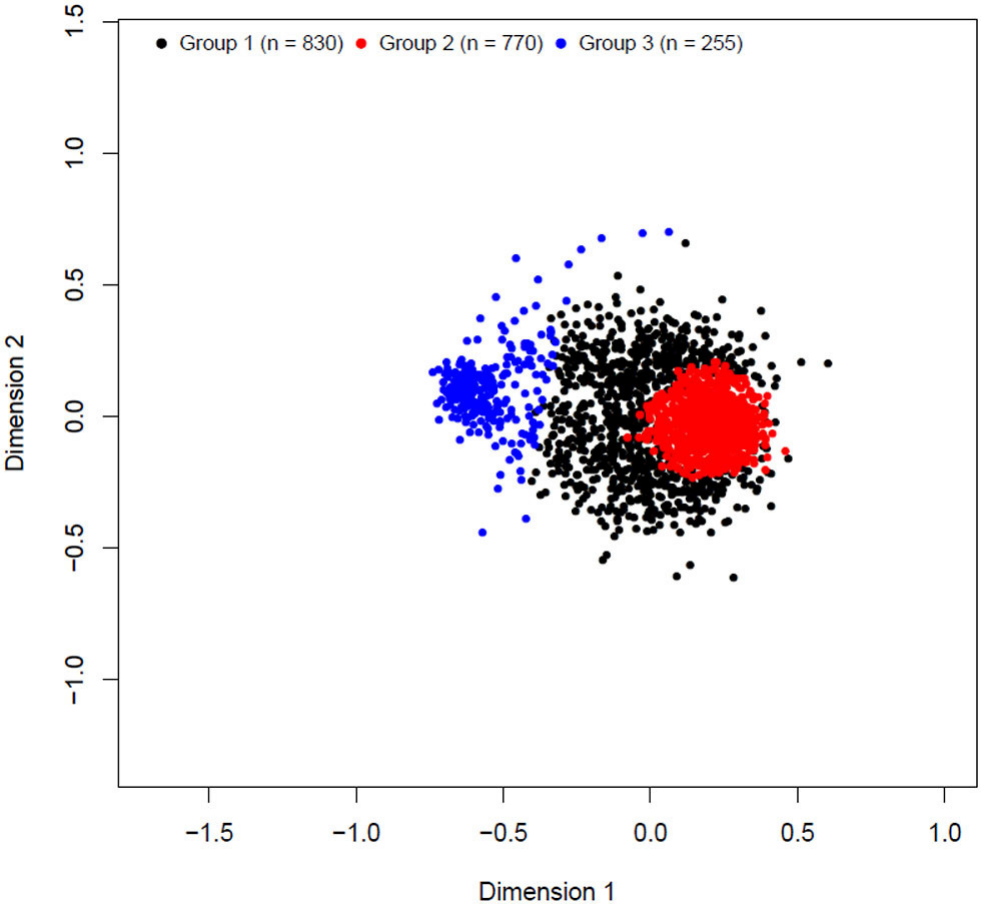
Multidimensional scaling bidimensional scatter plot of k-means clustering cross-validation groups.

The k-means clustering yielded three unbalanced groups with 830, 770, and 255 animals in groups 1, 2, and 3, respectively (Table 3). A multidimensional scaling bidimensional scatter plot according to the k-means groups is presented in Figure 1. Groups 1 and 2 were composed mainly by Braford breed with an average of 35% zebu contribution, whereas group 3 contained primarily Hereford breed (11% zebu contribution). As expected, the average genomic relationship was larger within than between groups. The average number of animals for the groups divided at random replicated 10 times was 618.33 animals (74.67 ± 6.18 Hereford, 12.33 ± 3.36 1/2 Braford, 496.67 ± 6.51 3/8 Braford, and 34.67 ± 3.51 1/4 Braford). Random groups had a similar average genomic relationship within groups (0.011 ± 0.045), and between groups, with the average close to zero.

**Table 3:** Number of individuals (N) and averages (±SD) of genomic relationship (*Gij*) within and between-group of Hereford breed and Braford composition breed for k-means clustering groups.

**Groups; *N***	**Hereford**	**Braford**			***Gij* within group**	***Gij* between group**
		**1/2^a^**	**3/8^a^**	**1/4^a^**		
1; 830	30	18	734	48	0.009 ± 0.035	0.000 ± 0.030
2; 770	30	18	696	26	0.054 ± 0.042	−0.008 ± 0.049
3; 255	164	1	60	30	0.070 ± 0.054	−0.003 ± 0.056

a *Zebu proportion*.

The accuracy of prediction for groups divided at random was higher than k-means clustering for groups 1 and 2 (Table 4). The groups generated by the k-means method had a larger number of crossbred animals, mainly in group 1 (800 animals of Braford breed, Table 3). For group 3, the accuracy of the predictions for k-means clustering and random methods was almost the same (0.3). Although, to our knowledge, there is no published genomic predictive study for *B. bovis* in cattle, the superiority of prediction accuracy using random clustering compared with k-means was also observed by Bock et al. (19) for tick resistance in Braford and Hereford cattle. Based on these results, the number of animals used for training the model is more important to achieve better prediction accuracy than the difference between the breed composition of group 3 and the other two groups. This is in agreement with a previous study that reported that a large number of animals in the training population is an important factor that improves the accuracy of genomic selection (18). The lower number of animals could explain the low accuracy for group 1 under k-means clustering in the training population and also the larger genetic relationship distance of groups 2 and 3. Accuracies of genomic predictions are related to the training data size and genetic relatedness between training and validation individuals (55, 56). Furthermore, the low IB heritability (Table 2) could be a determinant to the low prediction accuracies found in this study, as genomic selection reliability also depends on trait heritability (57). In this same population, Sollero et al. (58) found much higher accuracy for tick resistance (0.27 to 0.44), even when a specific SNP panel for TC with very low density was used. In dairy cattle, some traits related to resistance to infectious diseases have already been included in genetic evaluations and selection programs using genomic data (59), for example, the overall immune response (60, 61) that has higher heritability and prediction accuracy than results in the present study and the incidence of clinical mastitis (62) showing accuracies and heritability values for the predicted breeding values comparable with those found here. Other traits such as *Mycobacterium avium* subspecies paratuberculosis infection (63) or bovine respiratory disease in Holstein (64) also present low heritability and predictive accuracy. Despite low accuracy, genomic predictions could still be used as a viable tool to obtain a selection response for IB for replacement candidates. However, the expected genetic progress for this trait would be slow.

**Table 4:** Prediction accuracy of direct genomic value predictions for each k-means clustering and random cross-validation group using the ssGBLUP method for *Babesia bovis* infection level.

	**Prediction accuracy**		
	**Group 1**	**Group 2**	**Group 3**
k-means	0.180	0.225	0.346
Random	0.293	0.317	0.316

### Genome-Wide Association Studies for *Babesia bovis* Infection Level

**Figure 2:**
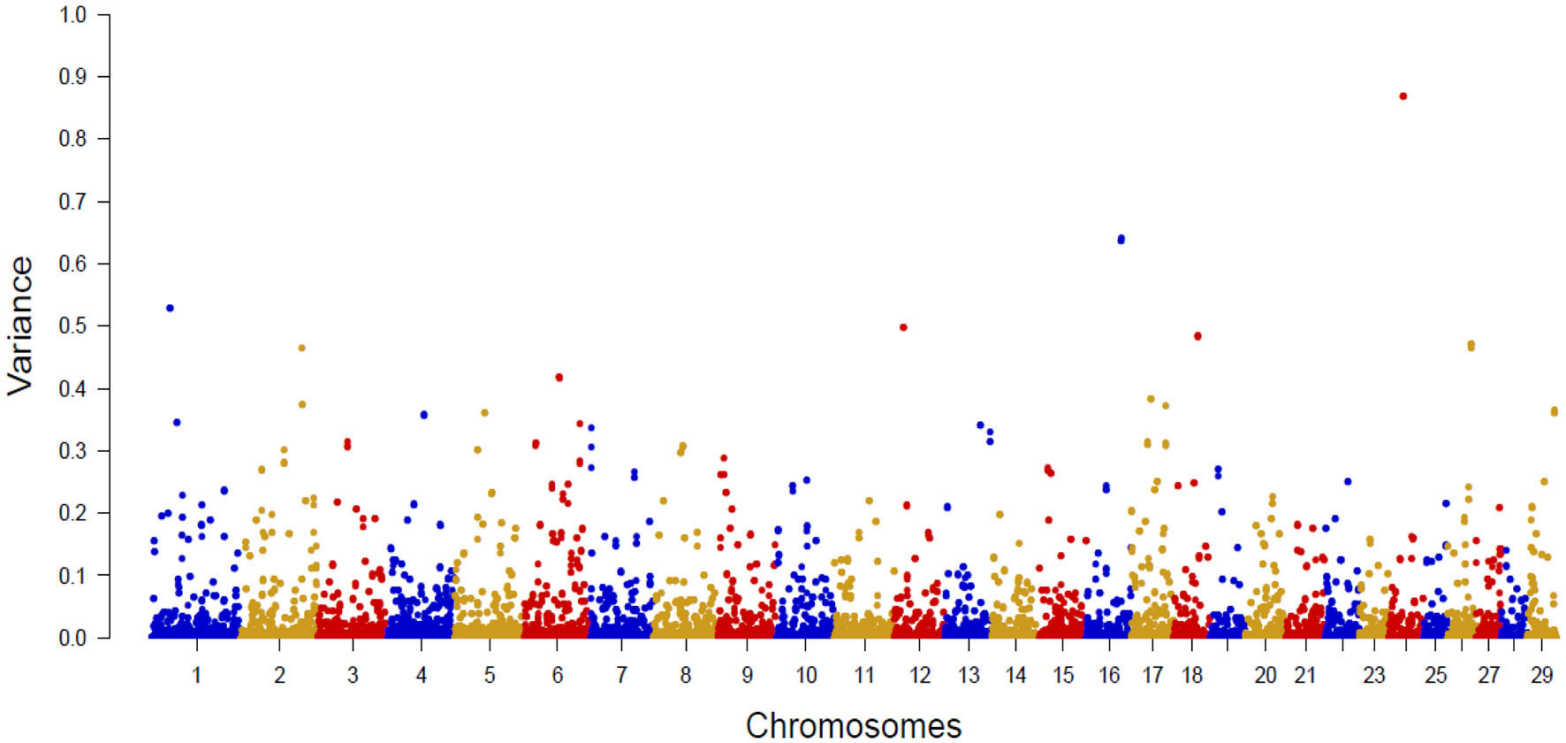
Manhattan plot of additive genetic variance explained by single SNP for *Babesia bovis* infection level.

Figure 2 shows the Manhattan plot with the percentages of additive genetic variance explained by each SNP for the IB trait. The top 10 SNPs (Table 5) explained 5.05% of IB additive genetic variance and identified 42 candidate genes involved in biological mechanisms that may underlie *B. bovis* resistance in cattle. Defense against parasites is mediated by sequential and coordinated immune responses called innate and adaptative (65), and several of the candidate genes participate in immune system pathways (*ATP8A1, LCP1, LRCH1, QSOX1, FGF2, DSC1, DSC3, FGFR2*, and *CEBPG*), which include adaptive and innate immune systems, and cytokine signaling pathways, indicating that genetic variations in these genes can alter the immune response and consequently, influence susceptibility and outcome of babesiosis in cattle. An essential aspect of *B. bovis* infection is that young calves have strong innate immunity, which lasts until about 6 months of age (66). Animals that survive infection with *B. bovis* become persistently infected and resistant to the clinical form of the disease, a phenomenon known as concomitant immunity (67). Adaptative immunity mechanisms are responsible for the absence of clinical signs in persistently infected animals.

**Table 5:** Description of the SNPs with the largest effects on *Babesia bovis* infection level in Hereford breed and Braford composition breed.

**BTA**	**SNP position**	**Genes^a^**	**Var (%)**
24	26,698,515	*DSC1, DSC2, DSC3*	0.853
16	62,734,784	*CEP350, QSOX1, LHX4, ACBD6, TOR1AIP1, TOR1AIP2, FAM163A*	0.640
1	32,883,377	*CADM2*	0.548
12	16,641,931	*LRCH1, ESD, HTR2A, RUBCNL, LRRC63, LCP1*	0.508
18	44,019,061	*PEPD, CEBPG, CHST8, KCTD15, SLC7A10, LRP3, WDR88, GPATCH1, FAAP24*	0.487
26	42,178,883	*ATE1, NSMCE4A, TACC2, BTBD16, FGFR2*	0.479
6	62,979,121	*ATP8A1, SHISA3, BEND4, SLC30A9, TMEM33, GRXCR1*	0.408
2	109,327,881	*Intergenic region*	0.377
17	34,752,485	*SPRY1, SPATA5, NUDT6, FGF2*	0.377
5	53,704,130	*SLC16A7*	0.367

a *Genomic coordinates for each gene based on the Bos taurus ARS.120 reference assembly were expanded by 500 kb upstream and downstream*.

*BTA, Bos taurus autosome; Var, proportion of additive genetic variance explained by the single SNP*.

LCP1 is a protein of the plastin family. This family is composed of actin-binding proteins that are conserved evolutionarily and expressed in different types of plants and animals (68). In mammals, three isoforms have been identified: T, I, and L-plastin. This latter group includes LCP1 that is expressed in hematopoietic cell lines, with essential functions in the activation of macrophages (69), lymphocytes, and granulocytes (70). According to Brown (71), the immune response against babesiosis depends on the activation of CD4+ T lymphocytes in the development of acquired protein antigen-specific responses. CD4+ T cells are essential for coordinating high-affinity IgG production and activating macrophages through the production of IFN-ɤ.

*LRCH1* also encodes proteins that influence the migration of CD4+ T cells (72). These cells play a regulatory role in the immune response and result in higher resistance to *R. microplus* in cattle, although other genes have been reported as mediators for T cell regulation (73, 74). Piper et al. (75) reported that Brahman animals (*B. indicus*) had higher percentages of T cells than did the Holstein–Friesians (*B. taurus*). Constantinoiu et al. (76) observed an increase of T cells in the skin around the site of *R. microplus* larvae attachment in both *B. indicus* and *B. taurus* cattle. Moré et al. (23) observed the participation of CD4+ T cell subtypes in Braford animals classified as tick-resistant. Another type of cell that influences the immune system is the B cell, which plays a role in the humoral immunity component of the adaptive immune system by secreting antibodies (77). The B cells are activated by the proteins encoded by *FGF2* and *FGFR2* genes through the signaling process, and *CEBPG* genes are involved in B cell differentiation. An increase of B lymphocytes in the dermis of tick-resistant cattle breeds was also observed, and differential B-lymphocyte regulation in lymph node tissue was associated with tick susceptibility (78).

The *FGF2* and *FGFR2* genes are involved in interleukins, fibroblast growth factor receptor, cytokine, and MAPK signaling pathways. The *SPRY1* gene also participates in fibroblast growth factor receptor and MAPK signaling pathways. Inflammatory interleukins, growth factors, and cytokines activate the MAPK signaling pathway, which regulates the immune response against intracellular parasites (79, 80). Moreover, cytokines stimulate natural killer cells to produce interferon-gamma (IFN-γ) during the chronic phase of *B. bovis* infection. The IFN-γ activates macrophages that synthesize and release nitric oxide, which inhibits *B. bovis* replication (81–84). The *CEBPG* gene also influences the natural killer cell process.

*DSC1, DSC2*, and *DSC3* are involved in the keratinization pathway and have been reported in tick resistance studies. Terminal differentiation of keratinocytes is important for the renewal of the stratum corneum, which plays an essential role in defense against the pathogen (85). According to the authors, tick bite lesions led to an increase of keratinocyte differentiation and the promotion of stratum corneum formation. Wang et al. (86) suggested that a dramatic reduction in keratin transcripts may occur in response to tick infestation. Also, *DSC1, DSC2, DSC3*, and *CADM2* genes participate in the cell adhesion process, which plays a critical role in initiating and sustaining the immune response against foreign pathogens (87). Piper et al. (88) reported that *DSC2* was detected as differentially expressed between tick-infested Holstein–Friesian and Brahman animals at the tick-attachment site. Moreover, a gene with an important biological function in controlling cellular adhesion and migration was associated with tick burden in cattle (89).

Genes involved in the hemostasis pathway, such as *SLC7A10* and *QSOX1*, were also found to harbor the regions identified as influencing IB. Sustained heavy *R. microplus* infestation has been shown to alter host hemostatic mechanisms by inhibiting platelet aggregation and coagulation functions (90). Several putative genes (*SPRY1, NUDT6, FGF2, FGFR2*, and *TACC2*) influencing IB participate in the cell proliferation process. In response to a heavy tick burden, many different types of cells proliferate to present exogenously derived antigens to the immune system (75). The authors identified genes differentially expressed between tick-infested Holstein–Friesian and Brahman animals that were involved in the cell proliferation process.

*HTR2A* is involved in inflammatory mediator regulation of transient receptor potential (TRP) channels and calcium signaling pathways. Modifications in intracellular calcium concentrations represent a fundamental mechanism in the control of inflammatory and immune cell functions (91). Intracellular calcium influx is a key process for lymphocyte activation and proliferation and cytokine synthesis (92, 93). Cytokine is involved in the immune process against *B. bovis* infection and replication, as discussed previously. As TRP channels favor intracellular calcium permeability, it is conceivable that, in association with other prominent molecular pathways, TRP channels could contribute to immune and inflammatory responses (91). Bagnall et al. (85) demonstrated that genes involved in the intracellular calcium regulation pathway are upregulated in response to cattle tick infestation in bovine skin. Also, the *HTR2A* gene participates in the ERK1 and ERK2 cascade process, which has control over inflammatory mediator synthesis and survival of innate immune cells (94).

## Conclusions

Predictive accuracies are related to the size of training populations, and despite its low heritability, genomic predictions could be used as a tool to improve genetics for *B. bovis* infection level in Hereford and Braford cattle. The effectiveness of this process would rely on generating a larger reference population than that used in the present study. Moreover, some candidate genes that participate in immunity system pathways were identified and could contribute to improving the genetic knowledge regarding *B. bovis* infection in cattle. Although the genetic correlation between *B. bovis* infection level and tick count was weak, some candidate genes for IB were also reported in tick infestation studies, and they were also involved in biological resistance processes.

## Data Availability Statement

The raw data cannot be made available because it is the property of the Braford and Hereford producers, Embrapa, and GenSys Consultants and this information is commercially sensitive.

## Ethics Statement

The animal study was reviewed and approved by Committee for Ethics in Animal Experimentation (CEEA) from the Federal University of Pelotas (Pelotas, RS, Brazil; process number 6849 and 9409). Written informed consent was obtained from the owners for the participation of their animals in this study.

## Author Contributions

LC: conception, analysis and interpretation of data, writing the draft, and approval of the version to be published. CB: analysis and interpretation of data and approval of the version to be published. RG and CO: sample preparation, laboratory analysis, interpretation of results, and approval of the version to be published. CG-G: experimental planning and design, data collection, sample preparation, and approval of the version to be published. AC: experimental planning and design, laboratory analysis, and critical revising of the manuscript. MO: technical and conceptual guidance, supervision of laboratory analysis, critical revising of the manuscript, and approval of the version to be published. FC: experimental planning and design, data collection, and approval of the version to be published. HO: experimental planning, critical revising of the manuscript, and approval of the version to be published. All authors contributed to the article and approved the submitted version.

## Conflict of Interest

The authors declare that the research was conducted in the absence of any commercial or financial relationships that could be construed as a potential conflict of interest.

## References

[1] McCosker PJ The global importance of babesiosis. In: Ristic M Kreier JP editors. Babesiosis. New York, NY: Academic Press (1981). p. 1–24.

[2] Nari A. Strategies for the control of one-host ticks and relationship withtick-borne diseases in South America. Vet Parasitol. (1995) 57:153–65. 10.1016/0304-4017(94)03117-F 7597780

[3] Grisi L Leite RC Martins JRS Barros ATM Andreotti R Cançado PHD. Reassessment of the potential economic impact of cattle parasites in Brazil. Rev Brasil Parasitol Vet. (2014) 23:150–6. 10.1590/S1984-29612014042 25054492

[4] Guglielmone AA. Epidemiology of babesiosis and anaplasmosis in Southand Central America. Vet Parasitol. (1995) 57:109–19. 10.1016/0304-4017(94)03115-D 7597777

[5] Oliveira-Sequeira TCG Oliveira MCS Araujo JP Amarante AFT. PCR-based detection of *Babesia bovis* and *Babesia bigemina* in their natural host *Boophilus microplus* and cattle. Int J Parasitol. (2005) 35:105–11. 10.1016/j.ijpara.2004.09.002 15619521

[6] Bock R Jackson L De Vos AJ Jorgensen W. Babesiosis of cattle. Parasitology. (2004) 129:247–69. 10.1017/S0031182004005190 15938514

[7] Hunfeld KP Hildebrandt A Gray JS. Babesiosis: recent insights into an ancient disease. Int J Parasitol. (2008) 38:1219–37. 10.1016/j.ijpara.2008.03.001 18440005

[8] Barros SL Madruga CR Araujo FR Menk CF Almeida MA Melo EPS. Serological survey of *Babesia bovis, Babesia bigemina* and *Anaplasma marginale* antibodies in cattle from the semi-arid region of Bahia, Brazil, by enzyme linked immunosorbent assays. Mem Inst Oswaldo Cruz. (2005) 100:613–7. 10.1590/S0074-02762005000600003 16302060

[9] Bock RE De Vos AJ Kingston TG Mclellan DJ. Effect of breed of cattle on innate resistance to infection with *Babesia bovis, B. bigemina* and *Anaplasma marginale*. Aust Vet J. (1997) 75:337–40. 10.1111/j.1751-0813.1997.tb15706.x 9196820

[10] Jonsson NN Bock RE Jorgensen WK. Productivity and health effects of anaplasmosis and babesiosis on *Bos indicus* cattle and their crosses, and the effects of differing intensity of tick control in Australia. Vet Parasitol. (2008) 155:1–9. 10.1016/j.vetpar.2008.03.022 18472219

[11] Buling A Criado-Fornelio A Acenzo G Benitez D Barbacarretero JC Florin-Cristensen M A quantitative PCR assay for the detection and quantification of *B. bovis* and *B. bigemina*. Vet Parasitol. (2007) 147:16–25. 10.1016/j.vetpar.2007.03.031 17466458

[12] Bilhassi TB Oliveira HN Ibelli AMG Giglioti R Regitano LCA Oliveira-Siqueira TCG. Quantitative study of *Babesia bovis* infection in beef cattle from São Paulo state, Brazil. Ticks Tick Borne Dis. (2014) 5:234–8. 10.1016/j.ttbdis.2013.11.002 24522252

[13] Giglioti R Oliveira HN Santana CH Ibelli AM Néo TA Bilhassi TB. *Babesia bovis* and *Babesia bigemina* infection levels estimated by qPCR in Angus cattle from an endemic area of São Paulo state, Brazil. Ticks Tick Borne Dis. (2016) 7:657–62. 10.1016/j.ttbdis.2016.02.011 26907097

[14] Giglioti R Oliveira HN Bilhassi TB Portilho AI Okino CH Marcondes CR. Estimates of repeatability and correlations of hemoparasites infection levels for cattle reared in endemic areas for Rhipicephalus microplus. Vet Parasitol. (2018) 250:78–84. 10.1016/j.vetpar.2017.12.010 29329629

[15] Benavides MV Sacco AM. Differential *Bos taurus* cattle response to *Babesia bovis* infection. Vet Parasitol. (2007) 150:54–64. 10.1016/j.vetpar.2007.08.022 17919816

[16] De Los Campos G Hickey JM Pong-Wong R Daetwyler HD Calus MPL. Whole-genome regression and prediction methods applied to plant and animal breeding. Genetics. (2013) 193:327–45. 10.1534/genetics.112.143313 22745228 PMC3567727

[17] Meuwissen THE Hayes BJ Goddard ME. Prediction of total genetic value using genome-wide dense marker maps. Genetics. (2001) 157:1819–29. Available online at: https://www.genetics.org/content/157/4/1819.full 11290733 10.1093/genetics/157.4.1819 PMC1461589

[18] Van Eenennaam AL Weigel KA Young AE Cleveland MA Dekkers JCM. Applied animal genomics: results from the field. Ann Rev Anim Biosci. (2014) 2:105–39. 10.1146/annurev-animal-022513-114119 25384137

[19] Cardoso FF Gomes CCG Sollero BP Oliveira MM Roso VM Picolli ML. Genomic prediction for tick resistance in Braford and Hereford cattle. J Anim Sci. (2015) 93:2693–705. 10.2527/jas.2014-8832 26115257

[20] Visscher PM Brown MA Mccarthy MI Yang J. Five years of GWAS discovery. Am J Hum Genet. (2012) 90:7–24. 10.1016/j.ajhg.2011.11.029 22243964 PMC3257326

[21] Barendse W Assessing Tick Resistance in a Bovine Animal for Selecting Cattle for Tick Resistance by Providing a Nucleic Acid From the Bovine Animal and Assaying for the Occurrence of a Single Nucleotide Polymorphism (SNP). Patent application WO2007051248-A1 1–146. Geneva: World Intellectual Property Organization (WIPO) (2010).

[22] Mapholi NO Maiwashe A Matika O Riggio V Bishop SC MacNeil MD. Genome-wide association study of tick resistance in South African Nguni cattle. Ticks Tick Borne Dis. (2016) 7:487–97. 10.1016/j.ttbdis.2016.02.005 26897394

[23] Moré DD Cardoso FF Mudadu M Malagó W Jr Gulias-Gomes CC Sollero BP. Network analysis uncovers putative genes affecting resistance to tick infestation in Braford cattle skin. BMC Genomics. (2019) 20:998. 10.1186/s12864-019-6360-3 31856720 PMC6923859

[24] Porto-Neto LR Jonsson NN D'Occhio MJ Barendse W. Molecular genetic approaches for identifying the basis of variation in resistance to tick infestation in cattle. Vet Parasitol. (2011) 180:165–72. 10.1016/j.vetpar.2011.05.048 21700395

[25] Porto-Neto LR Reverter A Prayaga KC Chan EKF Johnston DJ Bolormaa S. The genetic architecture of climatic adaptation in tropical cattle. PLoS ONE. (2014) 9:e113284. 10.1371/journal.pone.0113284 25419663 PMC4242650

[26] Turner LB Harrison BE Bunch RJ Porto Neto LR Li Y Barendse W A genome-wide association study of tick burden and milk composition in cattle. Anim Prod Sci. (2010) 50:235–45. 10.1071/AN09135

[27] Salem GH Liu X Johnsrude JD Dame JB Roman Reddy G. Development and evaluation of an extra chromosomal DNA-based PCR test for diagnosing bovine babesiosis. Mol Cell Probes. (1999) 13:107–13. 10.1006/mcpr.1998.0223 10208801

[28] Wharton RH Utech KBW The relation between engorgement and dropping of *Boophilus microplus* (canestrini) (ixodidae) to the assessment of tick numbers on cattle. J Aust Entomol Soc. (1970) 9:171–82. 10.1111/j.1440-6055.1970.tb00788.x

[29] Clayton D snpStats: SnpMatrix and XSnpMatrix Classes and Methods. R package version 1.18.0 (2014). Available online at: http://www.bioconductor.org/packages/release/bioc/html/snpStats.html (accessed October 6, 2016).

[30] Gianola D Rosa GJM. One Hundred years of statistical developments in animal breeding. Ann Rev Anim Biosci. (2015) 3:19–56. 10.1146/annurev-animal-022114-110733 25387231

[31] Aguilar I Misztal I Johnson DL Legarra A Tsuruta S Lawlor TJ. Hot topic: a unified approach to utilize phenotypic, full pedigree, and genomic information for genetic evaluation of Holstein final score. J Dairy Sci. (2010) 93:743–52. 10.3168/jds.2009-2730 20105546

[32] Legarra A Aguilar I Misztal I A relationship matrix including full pedigree and genomic information. J Dairy Sci. (2009) 92:4656–63. 10.3168/jds.2009-2061 19700729

[33] VanRaden PM. Efficient methods to compute genomic predictions. J Dairy Sci. (2008) 91:4414–23. 10.3168/jds.2007-0980 18946147

[34] Misztal I Tsuruta S Strabel T Auvray B Druet T Lee DW. BLUPF90 and related programs (BGF90). In: Proceedings of the 7th World Congress on Genetics Applied to Livestock Production. Montpellier: French Commune. (2002). p. 28–07.

[35] Gelman A Rubin DB Inference from iterative simulation using multiple sequences. Stat Sci. (1992) 7:457–72. Available online at: https://projecteuclid.org/download/pdf_1/euclid.ss/1177011136

[36] Geweke J Evaluating the accuracy of sampling-based approaches to the calculation of posterior moments. In: Bernardo JM Berger JO Dawid AP Smith AFM editors. Bayesian Statistic. New York, NY: Oxford University (1992). p. 625–31.

[37] Heidelberger P Welch PD Simulation run length control in the presence of an initial transient. Oper Res. (1983) 31:1109–44. 10.1287/opre.31.6.1109

[38] Plummer M Best N Cowles K Vines K Coda: convergence diagnosis and output analysis for MCMC. R News. (2006) 6:7–11. Available online at: https://cran.r-project.org/doc/Rnews/Rnews_2006-1.pdf#page=7

[39] R Core Team The R Project for Statistical Computing (2013). Available online at: http://www.R-project.org/ (accessed September 10, 2016).

[40] Saatchi M Schnabel RD Rolf MM Taylor JF Garrick DJ. Accuracy of direct genomic breeding values for nationally evaluated traits in US Limousin and Simmental beef cattle. Genet Select Evol. (2012) 44:38. 10.1186/1297-9686-44-38 23216608 PMC3536607

[41] Legarra A Robert-Granie C Manfredi E Elsen JM. Performance of genomic selection in mice. Genetics. (2008) 180:611–8. 10.1534/genetics.108.088575 18757934 PMC2535710

[42] Wang H Misztal I Aguilar I Legarra A Muir WM. Genome-wide association mapping including phenotypes from relatives without genotypes. Genet Res. (2012) 94:73–83. 10.1017/S0016672312000274 22624567

[43] VanRaden PM Van Tassell CP Wiggans GR Sonstegard TS Schnabel RD Taylor JF. Invited review: reliability of genomic predictions for North American Holstein bulls. J Dairy Sci. (2009) 92:16–24. 10.3168/jds.2008-1514 19109259

[44] Zhang Z Liu J Ding X Bijma P de Koning DJ Zhang Q Best linear unbiased prediction of genomic breeding values using a trait-specific marker-derived relationship matrix. PLoS ONE. (2010) 5:e12648 10.1371/journal.pone.0012648 20844593 PMC2936569

[45] Wickham H ggplot2: Elegant Graphics for Data Analysis. New York, NY: Springer (2009).

[46] Durinck S Spellman P Birney E Huber W. Mapping identifiers for the integration of genomic datasets with the R/Bioconductor package biomaRt. Nat Protoc. (2009) 4:1184–91. 10.1038/nprot.2009.97 19617889 PMC3159387

[47] Fabregat A Jupe S Matthews L Sidiropoulos K Gillespie M Garapati P. The reactome pathway knowledgebase. Nucleic Acids Res. (2018) 44:D649–55. 10.1093/nar/gkx1132 29145629 PMC5753187

[48] Biegelmeyer P Gulias-Gomes CC Roso VM Dionello NJL Cardoso FF Tick resistance genetic parameters and its correlations with production traits in Hereford and Braford cattle. Livest Sci. (2017) 202:96–100. 10.1016/j.livsci.2017.05.019

[49] Veerkamp RF Mulder HA Thompson R Calus MPL. Genomic and pedigree-based genetic parameters for scarcely recorded traits when some animals are genotyped. J Dairy Sci. (2011) 94:4189–97. 10.3168/jds.2011-4223 21787954

[50] Burrow HM Variances and covariances between productive and adaptive traits and temperament in a composite breed of tropical beef cattle. Livestock Prod Sci. (2001) 70:213–33. 10.1016/S0301-6226(01)00178-6

[51] Ayres DR Pereira RJ Boligon AA Baldi F Roso VM Albuquerque LG. Genetic parameters and investigation of genotype × environment interactions in Nellore × Hereford crossbred for resistance to cattle ticks in different regions of Brazil. J Appl Genet. (2015) 56:107–13. 10.1007/s13353-014-0238-5 25108748

[52] Budeli MA Nephawe KA Norris D Selapa NW Bergh L Maiwashe A Genetic parameter estimates for tick resistance in Bonsmara cattle. S Afr J Anim Sci. (2009) 39:321–7. 10.4314/sajas.v39i4.51125

[53] Berry DP Bermingham ML Good M More SJ. Genetics of animal health and disease in cattle. Ir Vet J. (2011) 64:5. 10.1186/2046-0481-64-5 21777492 PMC3102331

[54] Raszek MM Guan LL Plastow GS. Use of genomic tools to improve cattle health in the context of infectious diseases. Front Genet. (2016) 7:30. 10.3389/fgene.2016.00030 27014337 PMC4780072

[55] Habier D Fernando RL Garrick DJ. Genomic BLUP decoded: a look into the black box of genomic prediction. Genetics. (2013) 194:597–607. 10.1534/genetics.113.152207 23640517 PMC3697966

[56] Taylor JF Implementation and accuracy of genomic selection. Aquaculture. (2014) 420–21, S8–14. 10.1016/j.aquaculture.2013.02.017

[57] Hayes BJ Bowman PJ Chamberlain AJ Goddard ME. Invited review: genomic selection in dairy cattle – progress and challenges. J Dairy Sci. (2009) 92:433–43. 10.3168/jds.2008-1646 19164653

[58] Sollero BP Junqueira VS Gomes CCG Caetano AR Cardoso FF. Tag SNP selection for prediction of tick resistance in Brazilian Braford and Hereford cattle breeds using Bayesian methods. Genet Sel Evol. (2017) 49:49. 10.1186/s12711-017-0325-2 28619006 PMC5471684

[59] Chesnais JP Cooper TA Wiggans GR Sargolzaei M Pryce JE Miglior F. Using genomics to enhance selection of novel traits in North American dairy cattle. J Dairy Sci. (2016) 99:2413–27. 10.3168/jds.2015-9970 26778318

[60] Thompson-Crispi KA Sewalem A Miglior F Mallard B. Genetic parameters of adaptive immune response traits in Canadian holsteins. J Dairy Sci. (2012) 95:401–9. 10.3168/jds.2011-4452 22192219

[61] Thompson-Crispi KA Sargolzaei M Ventura R Abo-Ismail M Miglior F Schenkel F. A genome-wide association study of immune response traits in Canadian Holstein cattle. BMC Genomics. (2014) 15:559. 10.1186/1471-2164-15-559 24996426 PMC4099479

[62] Martin P Barkema HW Brito LF Narayana SG Miglior F. Symposium review: novel strategies to genetically improve mastitis resistance in dairy cattle. J Dairy Sci. (2018) 101:2724–36. 10.3168/jds.2017-13554 29331471

[63] Zare Y Shook GE Collins MT Kirkpatrick BW. Genome-wide association analysis and genomic prediction of *Mycobacterium avium* subspecies paratuberculosis infection in US Jersey Cattle. PLoS ONE. (2014) 9:e88380. 10.1371/journal.pone.0088380 24523889 PMC3921184

[64] Hoff JL Decker JE Schnabel RD Seabury CM Neibergs HL Taylor JF. QTL-mapping and genomic prediction for bovine respiratory disease in U.S. Holsteins using sequence imputation and feature selection. BMC Genomics. (2019) 20:555. 10.1186/s12864-019-5941-5 31277567 PMC6612181

[65] Abbas AK Lichtman AH Pober JS. Cellular and Molecular Immunology. Philadelphia: WB Saunders Company (2018).

[66] Goff WL Johnson WC Parish SM Barrington GM Tuo W Valdez RA. The age-related immunity in cattle to *Babesia bovis* infection involves the rapid induction of interleukin-12, interferon-gamma and inducible nitric oxide synthase mRNA expression in the spleen. Parasite Immunol. (2001) 23:463–71. 10.1046/j.1365-3024.2001.00402.x 11589775

[67] Brown WC Norimine J Knowles DP Goff WL Immune control of *Babesia bovis* infection. Vet Parasitol. (2006) 138:75–87. 10.1016/j.vetpar.2006.01.041 16510249

[68] Adams AE Shen W Lin C-S Leavitt J Matsudaira P. Isoform-specific complementation of the yeast sac6 null mutation by human fimbrin. Mol Cell Biol. (1995) 15:69–75. 10.1128/MCB.15.1.69 7799970 PMC231909

[69] Shinomiya H. Plastin family of actin-bundling proteins: its functions in leukocytes, neurons, intestines, and cancer. Int J Cell Biol. (2012) 2012:213492. 10.1155/2012/213492 22262972 PMC3259490

[70] Lin CS Aebersold RH Kent SB Varma M Leavitt J. Molecular cloning and characterization of plastin, a human leukocyte protein expressed in transformed human fibroblasts. Mol Cell Biol. (1988) 8:4659–68. 10.1128/MCB.8.11.4659 3211125 PMC365555

[71] Brown WC. Molecular approaches to elucidating innate and acquired immune responses to *Babesia bovis*, a protozoan parasite that causes persistent infection. Vet Parasitol. (2001) 101:233–48. 10.1016/S0304-4017(01)00569-6 11707299

[72] Xu X Han L Zhao G Xue S Gao Y Xiao J. LRCH1 interferes with DOCK8-Cdc42–induced T cell migration and ameliorates experimental autoimmune encephalomyelitis. J Exp Med. (2017) 214:209–26. 10.1084/jem.20160068 28028151 PMC5206493

[73] Domingues R Wohlres-Viana S Reis DR Teixeira HC Ferreira AP Guimarães SE. Expression of immune response genes in peripheral blood of cattle infested with *Rhipicephalus microplus*. Genet Mol Res. (2014) 13:4013–21. 10.4238/2014.May.23.12 24938612

[74] Jonsson NN Piper EK Constantinoiu CC. Host resistance in cattle to infestation with the cattle tick *Rhipicephalus microplus*. Parasite Immunol. (2014) 36:551–7. 10.1111/pim.12140 25313455

[75] Piper EK Jonsson NN Gondro C Lew-Tabor AE Moolhuijzen P Vance ME. Immunological profiles of *Bos taurus* and *Bos indicus* cattle infested with the cattle tick, *Rhipicephalus (Boophilus) microplus*. Clin Vaccine Immunol. (2009) 16:1074–86. 10.1128/CVI.00157-09 19474263 PMC2708397

[76] Constantinoiu CC Jackson LA Jorgensen WK Lew-Tabor AE Piper EK Mayer DG. Local immune response against larvae of *Rhipicephalus (Boophilus) microplus* in *Bos taurus indicus* and *Bos taurus Taurus* cattle. Int J Parasitol. (2010) 40:865–75. 10.1016/j.ijpara.2010.01.004 20109460

[77] Sasaki S Ito E Toki T Maekawa T Kanezaki R Umenai T. Cloning and expression of human B cell-specific transcription factor BACH2 mapped to chromosome 6q15. Oncogene. (2000) 19:3739–49. 10.1038/sj.onc.1203716 10949928

[78] Robbertse L Richards SA Clift SJ Barnard AC Leisewitz A Crafford JE. Comparison of the differential regulation of T and B lymphocyte subsets in the skin and lymph nodes amongst three cattle breeds as potential mediators of immune-resistance to *Rhipicephalus microplus*. Ticks Tick Borne Dis. (2018) 9:976–87. 10.1016/j.ttbdis.2018.03.034 29622516

[79] Soares-Silva M Diniz FF Gomes GN Bahia D. The mitogen-activated protein kinase (MAPK) pathway: role in immune evasion by trypanosomatids. Front Microbiol. (2016) 7:183. 10.3389/fmicb.2016.00183 26941717 PMC4764696

[80] Chen L Deng H Cui H Fang J Zuo Z Deng J. Inflammatory responses and inflammation-associated diseases in organs Oncotarget. (2017) 9:7204–18. 10.18632/oncotarget.23208 29467962 PMC5805548

[81] Shoda LK Palmer GH Florin-Christensen J Florin-Christensen M Godson DL Brown WC. *Babesia bovis*-stimulated macrophages express interleukin-1beta, interleukin-12, tumor necrosis factor alpha, and nitric oxide and inhibit parasite replication *in vitro*. Infect Immun. (2000) 68:5139–45. 10.1128/IAI.68.9.5139-5145.2000 10948137 PMC101760

[82] Goff WL Johnson WC Parish SM Barrington GM Elsasser TH Davis WC. IL-4 and IL-10 inhibition of IFN-gamma- and TNF-alpha-dependent nitric oxide production from bovine mononuclear phagocytes exposed to *Babesia bovis* merozoites. Vet Immunol Immunopathol. (2002) 84:237–51. 10.1016/S0165-2427(01)00413-5 11777537

[83] Goff WL Johnson WC Horn RH Barrington GM Knowles DP. The innate immune response in calves to *Boophilus microplus* tick transmitted *Babesia bovis* involves type-1 cytokine induction and NK-like cells in the spleen. Parasite Immunol. (2003) 25:185–8. 10.1046/j.1365-3024.2003.00625.x 12940961

[84] Goff WL Storset AK Johnson WC Brown WC. Bovine splenic NK cells synthesize IFNgamma in response to IL-12-containing supernatants from *Babesia bovis*-exposed monocyte cultures. Parasite Immunol. (2006) 28:221–8. 10.1111/j.1365-3024.2006.00830.x 16629708

[85] Bagnall N Gough J Cadogan L Burns B Kongsuwan K. Expression of intracellular calcium signalling genes in cattle skin during tick infestation. Parasite Immunol. (2009) 31:177–87. 10.1111/j.1365-3024.2008.01092.x 19292769

[86] Wang YH Reverter A Kemp D McWilliam SM Ingham A Davis CA Gene expression profiling of Hereford Shorthorn cattle following challenge with *Boophilus microplus* tick larvae. Aust J Exp Agric. (2007) 47:1397–407. 10.1071/EA07012

[87] Vestweber D. Adhesion and signaling molecules controlling the transmigration of leukocytes through endothelium. Immunol Rev. (2007) 218:178–96. 10.1111/j.1600-065X.2007.00533.x 17624953

[88] Piper EK Jackson LA Bielefeldt-Ohmann H Gondro C Lew-Tabor AE Jonsson NN. Tick-susceptible *Bos taurus* cattle display an increased cellular response at the site of larval *Rhipicephalus (Boophilus) microplus* attachment, compared with tick-resistant *Bos indicus* cattle. Int J Parasitol. (2010) 40:431–41. 10.1016/j.ijpara.2009.09.009 19852965

[89] Porto-Neto L Bunch R Harrison BE Prayaga K Barendse W. Haplotypes that include the integrin alpha 11 gene associated with tick burden in cattle. BMC Genet. (2010) 11:55. 10.1186/1471-2156-11-55 20565915 PMC2905322

[90] Reck J Berger M Jr Terra RMS Marks FS da Silva Vaz I Guimaraes JA Jr. Systemic alterations of bovine hemostasis due to *Rhipicephalus (Boophilus) microplus* infestation. Res Vet Sci. (2009) 86:56–62. 10.1016/j.rvsc.2008.05.007 18571684

[91] Parenti A De Logu F Geppetti P Benemei S. What is the evidence for the role of TRP channels in inflammatory and immune cells?. Br J Pharmacol. (2016) 173:953–69. 10.1111/bph.13392 26603538 PMC5341240

[92] Oh-hora M Rao A. Calcium signaling in lymphocytes. Curr Opin Immunol. (2008) 20:250–8. 10.1016/j.coi.2008.04.004 18515054 PMC2574011

[93] Feske S Skolnik EY Prakriya M. Ion channels and transporters in lymphocyte function and immunity. Nat Rev Immunol. (2012) 12:532–47. 10.1038/nri3233 22699833 PMC3670817

[94] Liu Y1 Shepherd EG Nelin LD. MAPK phosphatases–regulating the immune response. Nat Rev Immunol. (2007) 7:202–12. 10.1038/nri2035 17318231

[95] Cavani L Genetic study of Babesia bovis infection level and the association with tick resistance in Hereford and Braford cattle [dissertation]. São Paulo State University (Unesp), School of Agricultural and Veterinarian Sciences, Jaboticabal, Brazil (2019).

